# How newly diagnosed HIV-positive men who have sex with men look at HIV/AIDS – validation of the Chinese version of the revised illness perception questionnaire

**DOI:** 10.1186/s12879-017-2902-y

**Published:** 2018-01-02

**Authors:** Xiaobing Wu, Joseph T. F. Lau, Winnie W. S. Mak, Jing Gu, Phoenix K. H. Mo, Xiaodong Wang

**Affiliations:** 1 Department of STD control, Shenzhen Center for Chronic Disease Control, No.2021, Buxin Road, Luohu District, Shenzhen City, Guangdong Province People’s Republic of China; 2 Centre for Health Behaviors Research, School of Public Health and Primary Care, The Chinese University of Hong Kong, 5/F, School of Public Health, Prince of Wales Hospital, Shatin, NT Hong Kong, Special Administrative Region of China; 3 Department of Psychology, The Chinese University of Hong Kong, Shatin, NT Hong Kong, Special Administrative Region of China; 4 Department of Medical Statistics and Epidemiology, School of Public Health, Sun Yat-sen University, Guangzhou City, Guangdong province People’s Republic of China; 5 Chengdu Tongle Health Counselling Service Center, Chengdu City, Sichuan Province People’s Republic of China

**Keywords:** Illness perception, HIV infections, Men who have sex with men, Factor analysis, China

## Abstract

**Background:**

Newly diagnosed HIV-positive men who have sex with men (MSM) are an important subgroup in HIV intervention. How newly diagnosed HIV-positive MSM look at HIV/AIDS is consequential and is potentially associated with their risk behaviors and mental health problems. Illness representation has been used to define patients’ beliefs and expectations on an illness, and the revised Illness Perception Questionnaire (IPQ-R) has been developed to measure illness representations. This study aims to examine the psychometric properties of the IPQ-R among newly diagnosed HIV-positive MSM and to investigate their views towards HIV/AIDS.

**Method:**

A total of 225 newly diagnosed HIV-positive MSM completed the Chinese version of IPQ-R. Both confirmatory factor analysis (CFA) and exploratory factor analysis (EFA) were applied to examine the factor structure of IPQ-R.

**Results:**

CFA showed a poor goodness of fit to the original factor structure of IPQ-R. EFA of the IPQ-R revealed 7 factors, including Emotional Response, Treatment Control, Timeline-acute/chronic, Illness Coherence, Consequence, Personal Control and Helplessness. Cronbach’s alpha showed acceptable internal consistency for the derived factors, except the Personal Control (0.61) and Helplessness (0.55). Person correlation coefficients demonstrated that the derived factors of IPQ-R had significant associations with the outcome variables (depression and posttraumatic growth). The scores of the Emotional Response, Consequence, Treatment Control, Personal Control, Timeline-acute/chronic and Illness Coherence were above the midpoint, and the score of the Helplessness was below the midpoint.

**Conclusion:**

Both similarities and differences were found when the IPQ-R is applied to newly diagnosed HIV-positive MSM. The IPQ-R can be used with some refinements in future studies. Newly diagnosed HIV-positive MSM have a relatively high level of negative perceptions towards HIV/AIDS in both cognitive and emotional aspects.

## Background

HIV is a global public health problem with an estimated 36.7 million people living with HIV(PLHIV) and 2.1 million people newly infected with HIV in 2015 [1]. New HIV infection is associated with high infectivity due to the high viral loads detected in the first few months since the initial HIV infection occurs [2]. Newly diagnosed PLHIV, commonly defined as those who have been diagnosed as HIV positive for <12 months [3–6], is an important subgroup because a large proportion of them infect HIV recently and have high viral loads. Among those newly diagnosed HIV-positive men who have sex with men (MSM), about 30% to 60% are newly infected cases [7–9]. Such MSM have high prevalence of condomless anal intercourse and thus promote secondary HIV transmission [10]. Many of them have mental health problems as they encounter extreme difficulties when adjusting to their newly acquired HIV status [11]. How people look at the disease is considered to be associated with mental health problems and disease related behaviours [12]. Improvement of people’s view towards the disease can promote better health outcomes [13, 14].

Illness representation is used to define patients’ beliefs and expectations about an illness or somatic symptom [15, 16]. Cognitive representation and emotional representation are two types of inter-related illness representations. The Self-Regulatory Model (SRM, also known as the Illness Perception Model or Common Sense Model) specifies that health-related threats (a stimulus) would activate both cognitive and emotional representation processes (e.g. sadness, worry and anger), and such cognitive and emotional representations would affect the mode of coping strategies. The outcomes of the coping strategies would then be appraised, and the illness representation and subsequent coping strategies would be modified. The Illness Perception Questionnaire (IPQ) and the Revised Illness Perception Questionnaire (IPQ-R) have been developed to assess illness representation in different disease groups [17, 18]. By using the IPQ or IPQ-R, studies have shown that illness representation is associated with health-related outcomes among patients with acute or chronic diseases, such as self-care, service utilization, treatment adherence and mental health [12, 19, 20]. Improvement in illness representation among patients with a chronic disease can reduce mental health problems and increase health behaviours [21, 22].

China also faces an ongoing HIV epidemic and reported more than 0.50 million PLHIV at the end of 2014 [23]. Male-to-male sexual behaviour has become one main transmission route of HIV infection. The proportion of reported newly diagnosis HIV cases attributed to male-to-male sexual behaviour increased from 2.5% in 2006 to 25.8% in 2014 [23]. Among these newly diagnosed HIV-positive MSM, about 60% were considered to be infected with HIV recently [7]. At the moment of diagnosis and months after diagnosis, illness representation on HIV would be newly formed and more adjustable. Positive changes in illness representation on HIV may potentially facilitate positive attitude and emotion towards the disease, impacting coping strategies and quality of life. However, the IPQ-R for measuring PLHIV’s perception towards HIV infection has not been fully validated. In the present study, we investigated the psycho-metric properties of the Chinese IPQ-R, including the factor structure, internal consistencies, and correlations with some external variables (depression and posttraumatic growth) among newly diagnosed HIV-positive MSM in China, and further investigated how these MSM looked at the disease.

## Methods

### Study participants

Inclusion criteria were: 1) men who self-reported having had anal sex with at least one man in the last 12 months, 2) age 16 years old or above, and 3) being diagnosed as HIV positive in the last 12 months. Similar inclusion criteria of newly diagnosed HIV-positive MSM have been widely used in other published studies [3–6].

### Subject recruitment and data collection

The study was conducted in Chengdu, Sichuan province, a city located in Western China which has a population size of 10 million. The HIV prevalence among MSM in Chengdu was 15.5% in 2011 [24]. Recruitment was facilitated by a non-governmental organization (NGO) which had been providing HIV related services to MSM. Trained staff members of the NGO retrieved phone numbers, email addresses and QQ numbers (a popular electronic contact in China) from the service records of all HIV positive MSM who were potentially eligible to join this study. They then made attempts to contact these clients, screened their eligibility, briefed them about the purpose and logistics of the study, and invited them to participate in the study. Participants were explained clearly that refusals to join the study would not affect their opportunities to use any service and that they could quit anytime during the interview. Out of the 282 prospective participants contacted by the research team, 57 refused to join the study because they did not have time or had migrated to other cities (20.2%); 225 consented to join the study and completed the survey (79.8%).

Participants then visited the NGO. With written informed consent, they were face-to-face and anonymously interviewed by a well-trained peer interviewer in a private room. A structured questionnaire, which took about 25 min to complete, was used for the interview. Participants who completed the interview were given a cash incentive of 50 RMB (about $7.8 USD) to compensate them for their time. Ethics approval was obtained from the Survey and Behavioral Research Ethics Committee of the Chinese University of Hong Kong.

### Translation and modification of the IPQ-R

The original cognitive representation part of the IPQ-R has eight dimensions, including Identity (labeling the illness and identifying its symptoms), Cause (attributing likely causes of the illness), Timeline-acute/chronic (considering whether the illness is acute/chronic), Timeline-cyclical (considering whether the symptoms are fluctuating), Illness Coherence (overall comprehensibility of the illness), Consequence (assessing the severity of the illness), Personal Control (considering whether his/her illness is self-controlled) and Treatment Control (considering whether his/her illness can be controlled by treatment) [18]. The emotional representation part of the IPQ-R consists of six items (“when you think about having HIV, you get upset”, “having HIV makes you feel afraid”; “you get depressed when you think about having HIV”; “having HIV makes you feel anxious”; “having HIV makes you feel angry”; and “having HIV does not worry you”). The items of these subscales were presented in a mixed order in the questionnaire and were rated on 5-point Likert scales, ranging from 1 (strongly disagree) to 5 (strongly agree).

A panel consisting of three epidemiologists, two psychologists and one anthropologist was formed to adapt the content of the IPQ-R contextually for PLHIV. The modification made included: 1) The Timeline-cyclical subscale, which is important to illnesses that could not be ‘adequately captured within a simple acute/chronic dimension’ [18], was removed as new HIV infection is asymptomatic and is well known to be irreversible. 2) The causal attribution subscale was modified for HIV/AIDS - the eight items of Reynolds’s study on illness representation on PLHIV (i.e., carelessness, believed it could not happen to self, being deceived, not understanding the risk, chance/bad luck, god’s will, god’s punishment, and alcohol/drug use) [19] were used to replace the 18 items of the original scale which were not applicable to studying PLHIV (e.g. ageing, hereditary, family problems). 3) We also omitted the Identity subscale as HIV is asymptomatic among the majority of the newly diagnosed PLHIV. Similar omission of the identity dimension had also been reported in other IPQ studies among PLHIV [25] and non-PLHIV [26, 27].

A bilingual epidemiologist translated the modified English version of the IPQ-R into Chinese. The agreed version was back-translated into English by two other independent bilingual public health workers with doctorate degrees who worked on HIV prevention to ensure linguistic equivalence. In a pilot test, 11 HIV-infected MSM, including 8 being newly diagnosed, were interviewed with this modified Chinese version of the IPQ-R. Discussion and consensus were made to finalise the questionnaire.

### External variables for correlations

The 7-item validated Depressive Symptom Subscale of the 21-item Chinese Depression Anxiety Stress Scale (DASS-21) was used in the study, with responses ranging from 0 (‘did not apply to me at all’) to 3 (‘applied to me all the time’). The Cronbach’s alpha was 0.90. The Posttraumatic Growth (PTG) Scale was also used in this study [28, 29]. Two items related to spiritual matters (‘my understanding of spiritual matters’ and ‘my religious faith’) were removed due to incompatibility with the Chinese culture which de-emphasizes spirituality [30]. The remaining nine items formed a scale and were measured on a 5-point Likert Scale ranging from 1 (‘highly negative change’) to 5 (‘highly positive change’) [28, 29]. The Cronbach’s alpha was 0.87 in the present study.

### Statistical analyses

Confirmatory factor analysis (CFA), using the AMOS 7 software (Amos Development Corporation, 2010), was firstly performed for the items of the emotional representation and cognitive representation subscales (excluding items of the causal attribution subscale), to inspect whether the original model structure could be replicated in the present study [18]. The Minimum Discrepancy (CMIN), Goodness-of-Fit Index (GFI) and Root Mean Square Error of Approximation (RMSEA) were used to evaluate the goodness of fit.

Exploratory factor analysis (EFA) with varimax rotation would be conducted in case of poor goodness of fit of the CFA. The Kaisor-Meyer-Olkin (KMO) test and Bartlett’s sphericity tests were used to determine the suitability of conducting the EFA. Eigen-values (>1) and scree plots were used to determine the number of factors [18, 31, 32]. Items were removed if they failed to load significantly on a particular factor (0.4), or they loaded significantly (higher than 0.4) on two or more factors [32, 33]. An item would also be removed if it lacked conceptual coherence with the assigned factor. As cognitive representation and emotional representation are two parallel arms of the SRM, the two parts were analysed separately in the validation process of this study. EFA was applied separately to test the construct validity of the 28 items of the five original cognitive subscales (Timeline-acute/chronic, Consequence, Illness Coherence, Personal Control and Treatment Control subscales) and the 6-item Emotional Response subscale. Descriptive statistics of the subscale scores formed by the EFA were presented (e.g. ceiling effects and floor effects). Cronbach’s alpha coefficients were used to assess the internal consistency of the subscales. Pearson’s correlation coefficients were used to assess the correlations between each item and its respective factor, and between different factors of the IPQ-R. Data were analyzed using SPSS 16.0 for Windows; *p* < 0.05 was taken as statistically significant in the correlation analysis.

## Results

### Characteristics of the participants

The average age of the participants was 32.2 years (*SD* = 10.5 years); 53.8% were <=30 years old; 44.4% had attended colleges/universities;62.2% were employed full time; 48.0% had had a monthly income over 2000 RMB (about $312 USD). Two-thirds of them were single or divorced (66.7%), whilst 10.7% were cohabitating with a male partner and about one-fifth of them were currently married to a woman (21.8%). Of all the participants, 61.8% were diagnosed with HIV within the six months prior to the interview (average time since diagnosis = 5.6 months; *SD* = 3.8 months). About one-fifth (20.4%) of them had been receiving free antiretroviral treatment.

### CFA results

The results showed a poor goodness of fit, with CMIN = 1310.726 (*df* = 512, *p* < 0.001), GFI = 0.728 and RMSEA = 0.083. Therefore, EFA was applied to establish a new factor structure.

### EFA results

The EFA of the Emotional Response subscale identified a single factor which accounted for 67.8% of the total variance (KMO test = 0.89; Bartlett’s sphericity test = 874.4; *p* < 0.001). The item-subscale correlation coefficients of this subscale ranged from 0.46 to 0.84 (*p* < 0.05). The Cronbach alpha was 0.90.

The 28 items of five cognitive representation subscales (i.e., Timeline-acute/chronic, Consequence, Illness Coherence, Personal Control and Treatment Control) were subjected to EFA. Seven factors with eigen-value >1 were extracted, accounting for 58.8% of the total variance (KMO test = 0.81; Bartlett’s sphericity test =1971.4; *p* < 0.001). Four items were removed for the following reasons: two items showed factor loading <0.40 on all seven factors (‘There is nothing which can help your condition’ and ‘Your illness is a serious condition’); one factor (Factor 7) consisted only of a single item (‘You have a clear picture or understanding of your condition’ of the original illness coherence subscale); one item (‘Having HIV has serious financial consequences’ from the original consequence subscale) did not match with the interpretations of the other items of the same factor (Factor 2). The final EFA model therefore consisted of six factors (58.09% of the total variance; KMO test = 0.80; Bartlett’s sphericity test = 1558.09; *p* < 0.001).

The first four factors contained items that were similar to those in the original subscales and were named: 1) Treatment Control, 2) Timeline-acute/chronic, 3) Illness Coherence and 4) Consequence (Table 1). Their Cronbach’s alphas were respectively 0.76, 0.69, 0.76 and 0.70. The items of the original Personal Control Subscale were split into two subscales which were named ‘Personal Control’ (Factor 5) and ‘Helplessness’ (Factor 6); their Cronbach alpha values were respectively 0.61 and 0.55. The item-total correlation coefficients of the six identified factors ranged from 0.21 to 0.65.

**Table 1:** Exploratory factor analysis of items from the main cognitive representation dimensions among newly diagnosed HIV-positive MSM

	Factor 1	Factor 2	Factor 3	Factor 4	Factor 5	Factor 6	Original subscale
*Treatment Control*							
Anti HIV medication can control your condition	0.77	0.09	0.01	−0.08	0.22	−0.11	TC
Anti-HIV treatment will be effective in controlling your illness	0.74	−0.12	0.00	0.00	0.16	0.18	TC
Anti-HIV medication can control the progress of your HIV infection	0.73	0.12	−0.03	−0.03	−0.02	−0.24	TC
The negative effects of your illness can be prevented by anti HIV therapy	0.69	0.06	0.07	0.05	0.27	−0.02	TC
You have the power to influence the course of your condition	0.43	−0.23	0.13	−0.35	0.39	0.08	PC
*Timeline-acute/chronic*							
Your condition is likely to be permanent rather than temporary	0.18	0.72	0.00	0.09	0.09	−0.02	TA/C
You expect to have HIV for the rest of your life	0.25	0.72	−0.08	0.13	−0.12	0.06	TA/C
There is very little that can be done to improve your condition	−0.10	0.68	0.16	0.19	0.04	−0.02	TC
You will get ill when the time comes whether you are taking anti HIV medication or not	−0.20	0.49	0.25	0.23	−0.09	0.13	TC
Your condition will be in the same situation in a long time	0.16	0.45	−0.11	−0.11	0.42	0.20	TA/C
Your condition will improve in time	0.32	−0.58	0.15	−0.08	0.33	0.17	TA/C
*Illness Coherence*							
Having HIV does not make any sense to you	−0.09	−0.07	0.82	0.02	−0.03	0.11	IC
You don’t understand enough about HIV	0.11	0.07	0.77	0.07	−0.15	0.06	IC
The HIV virus is a mystery to you	0.02	0.06	0.67	0.18	0.21	0.10	IC
The symptoms of your condition are puzzling to you	0.12	0.06	0.58	0.39	0.10	0.23	IC
*Consequence*							
Having HIV has major consequences on your life	−0.10	0.27	0.30	0.72	0.04	0.00	CO
The fact that you have HIV causes difficulties for those who are close to you	0.12	0.01	0.13	0.72	0.05	0.12	CO
Having HIV strongly affects the way others see you	−0.03	0.28	0.17	0.57	0.01	0.08	CO
Having HIV does not have much effect on your life	0.16	−0.07	0.08	−0.65	0.25	0.18	CO
*Personal Control*							
There is a lot which you can do to control your condition	0.21	−0.22	0.08	−0.17	0.72	−0.09	PC
What you do can determine whether your condition gets better or worse	0.19	0.12	0.11	0.00	0.69	−0.30	PC
The course of your condition depends on you	0.28	0.01	−0.27	0.27	0.56	0.03	PC
*Helplessness*							
Nothing you do will affect your illness	−0.03	−0.02	0.15	0.03	−0.07	0.78	PC
Your actions will have no affect on the outcome of your condition	−0.08	0.09	0.17	0.03	−0.08	0.74	PC
Cronbach’s Alpha	0.76	0.69	0.76	0.70	0.61	0.55	
Initial Eigenvalues	4.11	3.61	2.52	1.46	1.18	1.07	
Cumulative % of variance explained	17.13	32.17	42.65	48.75	53.65	58.09	
Item-total correlation coefficients	0.42-0.64	0.21-0.53	0.53-0.59	0.43-0.65	0.34-0.46	0.38	

*TC* Treatment Control, *TA/C* Timeline-acute/chronic, *IC* Illness Coherence, *CO* Consequence, *PC* Personal Control

Deleted items included: “You have a clear picture or understanding of your condition” (Illness Coherence), “Your illness is a serious condition” (Consequence), “Having HIV has serious financial consequences” (Consequence), “There is nothing which can help your condition” (Personal Control)

An EFA extracted three factors among eight causal attribution items (59.27% of the total variance; KMO = 0.62; Bartlett’s test = 307.92; *p* < 0.001). The Cronbach alpha values were 0.86, 0.49, and 0.29, respectively (Table 2). As the internal consistency was too poor for Factor 2 and Factor 3 and as Factor 1 only consisted of two items, the causal attribution items were considered individually in the subsequent analyses.

**Table 2:** Exploratory factor analysis of items from the causal attribution dimension among newly diagnosed HIV-positive MSM

	Factor 1	Factor 2	Factor 3
God’s punishment	0.90	0.07	0.08
God’s will	0.89	0.10	0.20
Carelessness	−0.08	0.75	0.13
Did not understand the risk	0.23	0.61	−0.07
Believed it could not happen to self	0.39	0.55	−0.11
Being deceived	−0.09	0.49	0.46
Alcohol/drug use	0.07	−0.26	0.74
Chance/bad luck	0.23	0.24	0.65
Cronbach’s Alpha	0.86	0.49	0.29
Initial Eigen values	2.33	1.29	1.12
Cumulative % of variance explained	29.18	45.34	59.27
Item-total correlation coefficients	0.77	0.21-0.34	0.18

### Summary statistics and inter-correlations of the factors/items constructed

The mean scores and standard deviations for the derived factors/items are shown in Table 3. Except for a few items of causal attribution, the magnitudes of the floor effect (alcohol/drug use attribution: 25.3%) and the ceiling effect (e.g., carelessness attribution: 41.3%) were acceptable. Results of the descriptive statistics showed that the score of the Emotional Response, Consequence, Treatment Control, Personal Control, Timeline-acute/chronic and Illness Coherence were above the midpoint, and the score of Helplessness was below the midpoint. As for causal attribution, participants had the highest score on “carelessness”, followed by “believed it could not happen to self”. Other scores on causal attribution were below the midpoint and “alcohol/drug use” received the lowest score.

**Table 3:** Description of the derived factors/items of Chinese IPQ-R

Factors/items	No of items	Range of scores	Mean(SD)	Floor effects (%)^a^	Ceiling effects (%)^b^
Emotional Response	6	6-30	21.02(5.40)	1.33	2.67
Treatment Control	5	8-25	18.28(3.28)	0.44	5.33
Illness Coherence	4	4-20	12.25(3.21)	0.90	0.90
Timeline-acute/chronic	6	9-30	21.14(3.52)	0.44	0.44
Consequence	4	4-20	13.99(3.20)	0.44	4.44
Personal Control	3	4-15	10.17(2.19)	0.44	5.33
Helplessness	2	2-10	5.05(1.70)	6.67	1.78
Causal attribution					
God’s punishment	1	1-5	2.53(1.16)	16.89	8.44
God’s will	1	1-5	2.54(1.17)	18.22	8.89
Carelessness	1	1-5	4.06(1.04)	3.56	41.33
Did not understand the risk	1	1-5	2.95(1.26)	9.78	16.44
Believed it could not happen to self	1	1-5	3.46(1.19)	5.78	21.78
Being deceived	1	1-5	2.81(1.14)	8.00	12.00
Alcohol/drug use	1	1-5	1.99(0.87)	25.33	3.11
Chance/bad luck	1	1-5	3.12(1.19)	7.56	12.11

^a^The percentages of respondents scoring the minimum value of the subscales/items

^b^The percentages of respondents scoring the maximum value of the subscales/items

Correlation analysis showed that Emotional Response and Consequence were strongly correlated with each other (*r* = 0.75, *p* < 0.05; Table 4). These two factors were also both positively correlated with a number of causal attribution items (god’s will, god’s punishment, being deceived, did not understand the risk and chance/bad luck) and Timeline-acute/chronic (*r* ranged from 0.14 to 0.43, *p* < 0.05), and were negatively correlated with Treatment Control and Illness Coherence (*r* ranged from −0.16 to −0.46, *p* < 0.05). Illness coherence was also negatively associated with some causal attribution items (god’s will, god’s punishment, carelessness, being deceived and did not understand the risk), with *r* ranging from −0.14 to −0.38 (*p* < 0.05).

**Table 4:** Correlations matrix of the derived factors/items of Chinese IPQ-R^a^

		1	2	3	4	5	6	7	8	9	10	11	12	13	14	15
1	Emotional Response	1.00	−0.21^**^	−0.46^***^	0.38^***^	0.75^***^	−0.12	0.16^*^	0.43^***^	0.30^***^	0.03	0.15^*^	0.23^***^	0.25^***^	0.04	0.23^***^
2	Treatment Control		1.00	−0.06	−0.08	−0.16^*^	0.51^***^	−0.09	−0.23^***^	−0.17^*^	0.26^***^	0.07	−0.02	−0.01	−0.10	0.11
3	Illness Coherence			1.00	−0.12	−0.34^***^	−0.01	−0.30^**^	−0.23^***^	−0.23^***^	−0.22^***^	−0.38^***^	−0.26^***^	−0.14^*^	−0.09	−0.05
4	Timeline-acute/chronic				1.00	0.38^***^	−0.08	0.08	0.1	0.06	0.11	0.05	0.06	0.06	−0.18^**^	0.07
5	Consequence					1.00	−0.08	0.10	0.30^***^	0.22^***^	0.05	0.14^*^	0.23^***^	0.27^***^	−0.03	0.16^*^
6	Personal Control						1.00	−0.20^**^	−0.14^*^	−0.10	0.20^**^	0.16^*^	−0.07	0.10	0.08	0.06
7	Helplessness							1.00	0.12	0.07	0.06	0.15^*^	0.21^**^	−0.03	0.18^**^	0.11
	Causal attribution															
8	God’s punishment								1.00	0.76^***^	0.05	0.18^**^	0.25^***^	0.11	0.05	0.21^**^
9	God’s will									1.00	0.11	0.20^**^	0.27^***^	0.10	0.14^*^	0.29^***^
10	Carelessness										1.00	0.26^***^	0.20^**^	0.23^***^	−0.05	0.18^**^
11	Did not understand the risk											1.00	0.28^***^	0.11	0.02	0.09
12	Believed it could not happen to self												1.00	0.11	−0.04	0.18**
13	Being deceived													1.00	0.04	0.20^**^
14	Alcohol/drug use														1.00	0.18^**^
15	Chance/bad luck															1.00

^a^This analysis applied the bivariate correlation method. The Person correlation coefficients were shown in the table

*p < 0.05; **p < 0.01; ***p < 0.001

### Correlations between IPQ-R factors and external variables (depression and PTG)

Emotional representation was positively correlated with DASS depression (*r* = 0.60, *p* < 0.05) and negatively correlated with the PTG (*r* = −0.51, *p* < 0.05). All the four cognitive representation factors (Timeline-acute/chronic, Treatment Control, Illness Coherence and Consequence) were also significantly correlated with the DASS depression (*r* ranged from −0.24 to 0.48, *p* < 0.05) and PTG (*r* ranged from −0.39 to 0.23, *p* < 0.05). Personal Control was significantly correlated with PTG (*r* = 0.17, *p* = 0.01) but not with DASS depression (*r* = −0.13, *p* = 0.06), while Helplessness was not significantly associated with the two external variables (Table 5). In addition, three items of causal attribution (god’s will, god’s punishment and carelessness) were found to be significantly correlated with DASS depression (*r* ranged from −0.14 to 0.31, *p* < 0.05) and negatively correlated with PTG (*r* ranged from −0.26 to 0.15, *p* < 0.05). In addition, the item of ‘being deceived’ (attribution) was significantly associated with DASS depression (*r* = 0.21, *p* < 0.05) and the item of ‘chance/bad luck’ (attribution) was significantly associated with PTG (*r* = −0.13, *p* < 0.05).

**Table 5:** Correlations between IPQ-R subscales/items and external variables^a^

	Depression	Posttraumatic growth (PTG)
Emotional Response	0.60^***^	−0.51^***^
Treatment Control	−0.24^***^	0.23^**^
Illness Coherence	−0.34^***^	0.18^**^
Timeline-acute/chronic	0.14^*^	−0.17^*^
Consequence	0.48^***^	−0.39^***^
Personal Control	−0.13	0.17^*^
Helplessness	0.04	−0.11
*Causal attribution*		
God’s punishment	0.31^***^	−0.26^***^
God’s will	0.21^**^	−0.20^**^
Carelessness	−0.14^*^	0.15^*^
Did not understand the risk	0.14^*^	−0.08
Believed it could not happen to self	0.01	−0.11
Being deceived	0.21^**^	−0.04
Alcohol/drug use	−0.03	<0.01
Chance/bad luck	0.13	−0.13^*^

^a^This analysis applied bivariate correlation to analyze the data and Person correlation coefficients were shown in the table

*:*p* < 0.05; **:*p* < 0.01; ***:*p* < 0.001

## Discussion

A number of studies have investigated the factor structure of the IPQ-R among different kinds of patients [32, 34–36]. However, as far as we know, this is the first study to fully validate the IPQ-R structure among PLHIV in mainland China. The validation results allow us to use the IPQ-R for measuring PLHIV’s perceptions on HIV infection in future studies, and it also provides suggestions on the development of illness perception interventions which may promote PLHIV to have better health outcomes.

In this study, we have made some modifications in the validation process similar to other studies [25, 35, 36]. It is common for researchers to delete some items and/or subscales of IPQ-R for contextual reasons. Hagger and colleagues have removed the Timeline-cyclical subscale as it is deemed irrelevant to patients who have been diagnosed with a condition that was largely asymptomatic [35]. Other researchers have changed items of the Identity subscale [36], removed items related to the Cause and Identity subscales [25–27, 37], and changed wordings of some items [26, 32]. Our practice was therefore appropriate.

The factor structure of the four cognitive representation subscales (Factors 1 to 4: Treatment Control, Illness Coherence, Timeline-acute/chronic and Consequence) and the Emotional Response subscale in this study are highly similar to those identified in the previous studies [32]. Their Cronbach alpha values are acceptable (i.e., 0.7 or above). These five dimensions are also significantly associated with the two external variables (depression and PTG). We therefore suggest the use of these subscales in future studies to measure illness representation of HIV/AIDS. The original subscale on Personal Control is split into two new subscales (Factor 5 and Factor 6: Personal Control and Helplessness) and low internal reliabilities are observed. Consistently, other researchers have reported similar problems with the Control subscale [32]. They suggest that the importance of control might vary across culture [38], and that respondents may be confused about the wordings of several items of the subscale, especially those with negative wordings, such as ‘*nothing* you do will affect the illness’ and ‘there is *nothing* which can help your condition’ [38, 39]. Other researchers have commented that such negative wordings may be ambiguous and confusing [40]. The Personal Control and Treatment Control subscales for HIV/AIDS are conceptually important; our results also show significant correlations between Treatment Control and the two external variables. However, we have not found satisfactory internal consistency, particularly for the Personal Control subscale. Further studies are required before we are confident to use these subscales in studies on HIV-related illness representations.

The original items of the subscale on causal attribution (e.g. ageing, hereditary, family problems) are not applicable to HIV/AIDS. Reynolds et al. attempt to develop items that are relevant to HIV infection [19]. In their study, common attributions include ‘carelessness’, ‘believed it could not happen to me’, ‘not understanding the risk’, ‘chance/bad luck’ and ‘being deceived’. The current study follows Reynolds’s suggestion and uses their items. The EFA results for these items showed a clear factor structure but low internal consistency. A lot of MSM believe that HIV infection will not happen to them as they have little knowledge about local HIV epidemic and are not aware of the serostatus of their partners. Alcohol use or drug use are widely considered to be associated with risky sexual behaviours and HIV infection [41]. However, only a small proportion of newly diagnosed MSM considered it as the cause of HIV infection. This finding suggests that what we find from the research may be varied from what newly diagnosed MSM think. Future studies are needed to explore the attribution of HIV among MSM and to see whether their attribution differs from those who acquired HIV from other routes, and from those who have been diagnosed for a longer period of time.

Consistent with previous work [18], the derived factors/items of IPQ-R for newly diagnosed HIV-positive MSM reveal some inter-relationships which are consistent with the existing literature. Negative beliefs of illness coherence, timeline-acute/chronic, consequence, and treatment control of HIV infection are related to negative emotional responses, implying potential links between cognitive and emotional representations. Leventhal et al. have postulated the internal relationships inside the SRM and assumed that peoples’ emotional representation towards a disease would influence their cognitive representation towards the disease, which finally affects their coping measures and health outcomes [42]. The present study supports such notions and suggests that future studies are required to explore the relationship between cognitive and emotional representation, and their impact on coping and health related outcomes for better understanding of the SRM.

The scores of items and the derived subscales demonstrate that newly diagnosed HIV-positive MSM have negative perceptions toward HIV infection. They have a relatively high level of negative emotions towards HIV infection and consider HIV infection would bring serious consequences to them. On the contrary, they only score about midpoint in illness coherence, indicating that they have little knowledge of the disease. Such negative perceptions, according to the theory of SRM, may lead to negative or passive coping strategies and poor health outcomes. Newly diagnosed HIV-positive MSM are in the formative stage of their perceptions towards HIV/AIDS. Interventions to foster more positive changes on illness perceptions are warranted.

This study has some limitations. First, the study is conducted among newly diagnosed HIV-positive MSM whose characteristics may be different from those who have been diagnosed for a longer period of time, or those who are infected with HIV through other routes. Attention should be paid when using the derived subscales/items in other PLHIV subgroups. Second, the internal reliability of some factors (e.g., Personal control and Helplessness) is below satisfactory, and modifications of the items from these factors seem necessary. Third, about one fifth of eligible subjects refuse to participate in this study. Although the characteristics of this subgroup are unknown, they may be less likely to seek healthcare services or social support, and may have even more negative perceptions towards HIV/AIDS. Selection bias might therefore exist.

## Conclusions

In conclusion, this study refines the factor structure of the IPQ-R among newly diagnosed HIV-positive MSM in mainland China. Both similarities and differences are found when comparing the modified factor structure with the original one. Future studies may investigate the applicability of this questionnaire to other HIV positive MSM with longer periods of diagnosis and to other PLHIV. With the refined instruments, future studies may better discern relationships between how PLHIV look at HIV/AIDS and the health outcomes.

## Abbreviations

CFA: Confirmatory factor analysis
CMIN: The minimum discrepancy
DASS: Depression anxiety stress scale
EFA: Exploratory factor analysis
GFI: Goodness-of-Fit Index
IPQ: The Illness Perception Questionnaire
IPQ-R: The revised Illness Perception Questionnaire
KMO: Kaisor-Meyer-Olkin
MSM: Men who have sex with men
NGO: Non-governmental organization
PLHIV: People living with HIV
PTG: Posttraumatic Growth
RMSEA: Root Mean Square Error of Approximation
SRM: The self-regulatory model
